# Regulatory effect of Dimethyl Sulfoxide (DMSO) on astrocytic reactivity in a murine model of cerebral infarction by arterial embolization

**Published:** 2013-03-30

**Authors:** Catalina Lapuente Chala, Carlos Augusto Rengifo Valbuena, Marco Fidel Ávila Rodríguez, Angel Céspedes Rubio

**Affiliations:** a Department of Animal Health-Faculty of Veterinary Medicine & Zootecnia Universidad del Tolima, E-mail: catalinalapuente@yahoo.com; b Pathology Department of Animal Health - Faculty of Veterinary Medicine & Zootecnia Universidad del Tolima, E-mail: carlosa_rengifo@hotmail.com; c Departament of Biology Science Faculty Universidad del Tolima, E-mail: carlosa_rengifo@hotmail.com; d Toxicology Laboratory. Department of Animal Health Medical Faculty Veterinary Medicine & Zootecnia Universidad del Tolima, E-mail: aecesped@ut.edu.co; e Group for Research in Neurodegenerative Diseases.

**Keywords:** Brain cerebral ischemia, dimethyl sulfoxide (DMSO), immunohistochemistry, astrocytes, neuroglia gliosis

## Abstract

**Introduction:**

The pathophysiology of cerebral ischemia is essential for early diagnosis, neurologic recovery, the early onset of drug treatment and the prognosis of ischemic events. Experimental models of cerebral ischemia can be used to evaluate the cellular response phenomena and possible neurological protection by drugs.

**Objective:**

To characterize the cellular changes in the neuronal population and astrocytic response by the effect of Dimethyl Sulfoxide (DMSO) on a model of ischemia caused by cerebral embolism.

**Methods:**

Twenty Wistar rats were divided into four groups (n= 5). The infarct was induced with α-bovine thrombin (40 NIH/Unit.). The treated group received 90 mg (100 μL) of DMSO in saline (1:1 v/v) intraperitoneally for 5 days; ischemic controls received only NaCl (placebo) and two non-ischemic groups (simulated) received NaCl and DMSO respectively. We evaluated the neuronal (anti-NeuN) and astrocytic immune-reactivity (anti-GFAP). The results were analyzed by densitometry (NIH Image J-Fiji 1.45 software) and analysis of variance (ANOVA) with the Graph pad software (Prism 5).

**Results:**

Cerebral embolism induced reproducible and reliable lesions in the cortex and hippocampus (CA1)., similar to those of focal models. DMSO did not reverse the loss of post-ischemia neuronal immune-reactivity, but prevented the morphological damage of neurons, and significantly reduced astrocytic hyperactivity in the somato-sensory cortex and CA1 (*p* <0.001).

**Conclusions:**

The regulatory effect of DMSO on astrocyte hyperreactivity and neuronal-astroglial cytoarchitecture , gives it potential neuroprotective properties for the treatment of thromboembolic cerebral ischemia in the acute phase.

## Abstract (es)

**Introducción:**

La fisiopatolog ía de la isquemia cerebral es fundamental para el diagnóstico oportuno, la recuperación neurológica, la instauración temprana del tratamiento y el pronóstico del evento isquémico. Los modelos experimentales de isquemia cerebral, permiten evaluar los fenómenos de respuesta celular y la posible neuroprotección por fármacos.

**Objetivo:**

caracterizar los cambios celulares en la población neuronal y la respuesta astrocitaria por efecto del dimetilsulfóxido (DMSO) en un modelo de isquemia por embolia cerebral. Métodos: Se utilizaron 20 ratas Wistar distribuidas en cuatro grupos (n =5). Se indujo embolia cerebral con α- trombina bovina (40 NIH/Unit.) y el grupo tratado recibió 90 mg (100 μL) de DMSO en NaCl (1:1 v/v) intraperitoneal por 5 días; el control isquémico, recibió solamente NaCl (placebo) y los dos grupos no isqu émicos (simulados) recibieron NaCl y DMSO respectivamente. Se evaluó la inmunoreactividad neuronal (anti-NeuN) y astrocitaria (anti-GFAP). Los resultados fueron analizados por densitometría (Fiji-Image J NIH 1.45 software) y análisis de varianza (ANOVA) con el programa Graph pad Prism 5.

**Resultados:**

La embolia cerebral produjo lesiones reproducibles y confiables en corteza e hipocampo (CA1), similares a las de los modelos focales. El DMSO no revirtió la pérdida de la inmunoreactividad neuronal postisquemia, pero previno el daño morfológico neuronal y redujo significativamente la hiperreactividad astrocitaria en CA1 y corteza somatosensoria (*p *<0.001).

**Conclusiones:**

El efecto regulatorio del DMSO sobre la hiperreactividad astrocitaria y la citoarquitectura neuronal - astroglial, le confiere características potencialmente neuroprotectivas para el tratamiento de la isquemia cerebral tromboembólica en fase aguda.

## Introduction

Cerebrovascular disease (CVD) is the leading cause of permanent disability and the third leading cause of death in Colombia and in the world ^1^ ^,^ ^2^. Cerebral ischemia represents 85% of CVD, causing about 6,000,000 deaths per year^3^. CVD causes cellular death by various mechanisms^2^ ^,^ ^4^, with obstructive type ischemia being the most frequent, caused by a clot that occludes one or more branches of a major artery, usually the middle cerebral artery (MCA)^2^. The models of ischemia in rats replicate well the lesions described in humans, given their anatomical and hemodynamic similarities in cerebral circulation^5^. Additionally, the multifocal cerebral emboli model allows evaluation of the clinical utility of thrombolytic agents and other pharmaceuticals^4^.

During cell death in the acute phase (less than 3 days) astrocytic pathology is not evidenced and in the sub-acute phase (greater than 3 days) there is progressive damage and cell loss^6^ with the neurons in the hippocampus (CA1), layers III-V cortical and striatum being more susceptible^3^ ^,^ ^7^.

Astrocytes are competent cells of the innate immune system involved in primary recognition of harmful signals^8^. They provide neurotrophic factors that promote neuronal survival^7^participate in the uptake and metabolism of glutamate, aid the antioxidant capacity of the neurons and support synaptogenesis and neurogenesis, indicating close neuron-astrocyte communication^9^.

The clinical management of cerebral ischemia is focused on the administration of drugs to restore blood flow and possibly reverse neurological deterioration^1^; however, despite multiple drugs being studied, the activator of tissue plasminogen is the only drug approved by the FDA for use in humans, thus necessitating a search for new alternative therapies.

Dimethyl Sulfoxide (DMSO) has been used in biological research as a solvent of hydrophobic substances ^14^ and used successfully in the treatment of rheumatic, musculoskeletal, dermatological, gastrointestinal and urinary tract disorders for its anti-inflammatory and antioxidant properties^10^. Since DMSO can cross the blood-brain barrier (BBB), it has been used to treat cerebral edema; it has potential neuroprotective actions, such as the capture of free radicals and inhibition of prostaglandin and vasodilatation receptors, and it may potentiate the effect of NSAIDs and significantly reduce the infarct volume ^11^. However, some side effects have been reported that are possibly dose dependent or stem from an electrolyte imbalance in chronic treatments ^10^ ^,^ ^11^.

The purpose of this study was to characterize neuronal changes and the subsequent astrocytic response to cerebral ischemia produced by arterial embolism in Wistar rats treated with DMSO.

##  Materials and Methods 

### Animals:

Twenty male Wistar rats with an average weight of 200 g were used. They were maintained in a proper animal facility, according to the rules set forth in Act 84 of 1989 and described in the "Guide for the Care and Use of Laboratory Animals" for the protection and management of laboratory animals. Experiments were conducted in the animal facility of the School of Science at the University of Tolima upon approval of the Local Ethics Committee.

### Surgical procedure:

The intervention on the animals occurred under general anesthesia with a mixture of xylazine 2% (10 mg/kg), plus 5% ketamine (90 mg/kg) intraperitoneally (ip) and atropine 1:1,000 (0.1 mg/kg) subcutaneously (sc). The temperature was monitored during surgery. We used a multifocal model of ischemia for cerebral emboli ^12^ ^-^ ^15^ with some modifications described as follows: the common carotid arteries (CCA), internal carotid artery (ICA) and external carotid artery (ECA) were exposed; blood flow was temporarily suspended and the posterior occipital branches, the ascending larynx and superior thyroid branches were cauterized by ACE (Electrocautery Bovie Aaron Medical, HIT1 USA), and distally attached (polypropylene 6/0 Prolene (r), USA) creating a trunnion through which a vascular catheter was introduced (ETFE-NIPRO-24G) loaded with 4 μL (40 NIH/U) of α bovine thrombin (α-tb HTI Lab, BCT1020), advancing it to the ACM. Ten μl of blood were drawn into the catheter and after 5 min a clot was obtained, which was suspended in 100 μL of saline solution and slowly injected. The catheter was removed and the ACE was ligated (Ethilon(r) nylon 6/0). Circulation was restored and closing was in one plane (Ethilon(r) nylon 4/0).

### Experimental Design:

The animals were divided into two experimental groups (n= 5 each) and two controls (n= 5 each). Cerebral embolism was induced with α-tb in the rats in experimental group 1. They were injected daily with 200 μL of NaCl 0.9% N (placebo) ip for 5 days. The animals in the experimental group 2 were embolized with α-tb and treated with 90 mg (100 μL) of 90% DMSO (Synthesis, Lab) diluted in 100 μl 0.9% NaCl N (1:1. v:v) ip daily for five days. For the rats in control group 1 no cerebral ischemia was performed, the procedure was simulated (sham) and they received NaCl (placebo). The rats in control group 2 also had a simulated ischemia (sham) and received treatment with DMSO at the same dosage, interval and duration as experimental group 2.

Eight days after the last treatment, the animals were anesthetized with sodium pentobarbital 60 mg/kg (Penthal 6.48%, Invet, SA) and xylazine 10 mg/kg (Rompun(r) 2% Bayer SA) administered intracardiacally and using aortic advance at moderate positive pressure (Syringe and infusion valve BD x 50 cc) with NaCl 0.9% N (200 mL) and subsequently fixed with paraformaldehyde (PFA) 4% (200 mL). The brains were extracted and post-fixed (PFA 4% at 4° C/48 h) for subsequent cutting into coronal sections of 50 microns (Vibratome 1500) and conservation in a cryopreservative. As a marker of glial activity an antibody against the glial fibrillary acidic protein (anti-GFAP) was used , while the neuronal antinuclear antibody (Anti-NeuN) was used as a marker of neurodegeneration.

Conventional immunohistochemistry was carried out following the protocol described in Current Protocols in Neuroscience^16^ with modifications as follows: Inhibition of endogenous peroxidase (Methanol:PBS 1:1 - 1% H_2_O_2_), washed with PB 0.1 M, pre-incubation (PB 0.1M -Triton 100X 3% - BSA 1%) for 60 minutes and incubation at 4°C overnight in the primary antibodies (anti-NeuN and anti-GFAP) prepared in buffer (PB 0.1 M-Triton 100X0.3 % and BSA 0.3%). Consecutive washes with PB 0.1 M and incubation in secondary antibody (goat anti-mouse and goat anti-rabbit 1:500) for 2 h at room temperature. Incubation in Avidin/Biotin (1:250 each) for two hours and developed with diaminobenzidine (11 mg/15 mL PB 0.1 M -H_2_O_2_ 0.02%). The sections were put on slides, covered with transparent slips and sealed with Consult Mount.

Digitalized images were taken in all cases in the cortical laminates III to V of the primary somatosensory cortex and in the CA1 area of the ipsilateral hippocampus (10X) and then subjected to study by densitometry with the Fiji-Image J program (v-1.45 - NIH). Statistical analyses were carried out using ANOVA and multiple comparisons between treatment means (Tukey), upon proof of homogeneity of variances and normality test. Data were analyzed using GraphPad (Prism v-5.0).

## Results

**Figure 1:**
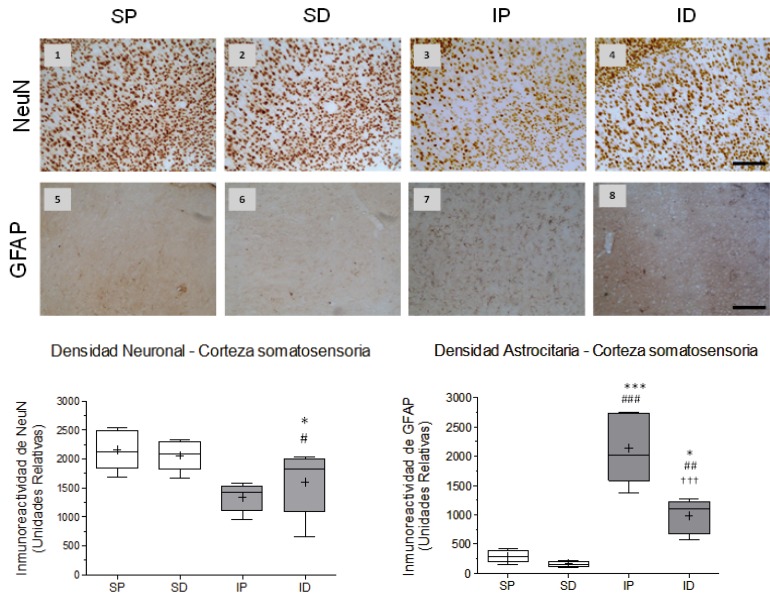
Effect of cerebral embolism and DMSO treatment on neuronal and astrocytic reactivity in the somatosensory cortex. A) Representative images of neuronal nuclear immune reactivity (NeuN) (1-4) and of astrocytic (GFAP) immune reactivity (5-8) from the groups: SP Sham + Placebo, SD: Sham + DMSO, IP: Ischemia + Placebo , ID: ischemia + DMSO. B) B) Statistics of the immune reactivity of NeuN in cerebral cortex. Significant differences between the IP/SP groups (* p <0.05) and IP/SD groups (# p <0.05) were observed. No differences were found due to the effects of DMSO treatment with respect to any of the groups. C) Statistics of immune reactivity of GFAP in the ipsilateral cortex. Highly significant differences were found between groups IP/SP (*** p <0.001), IP/SD (# # # p <0.001) and ID/IP († † † p <0.001). In addition, significant differences were observed between the ID/SP groups (* p <0.05) and highly significant differences between the ID group and its SD control (# # p <0.01). Data are expressed as the mean ± SEM. Scale bar = 50 microns

**Figure 2:**
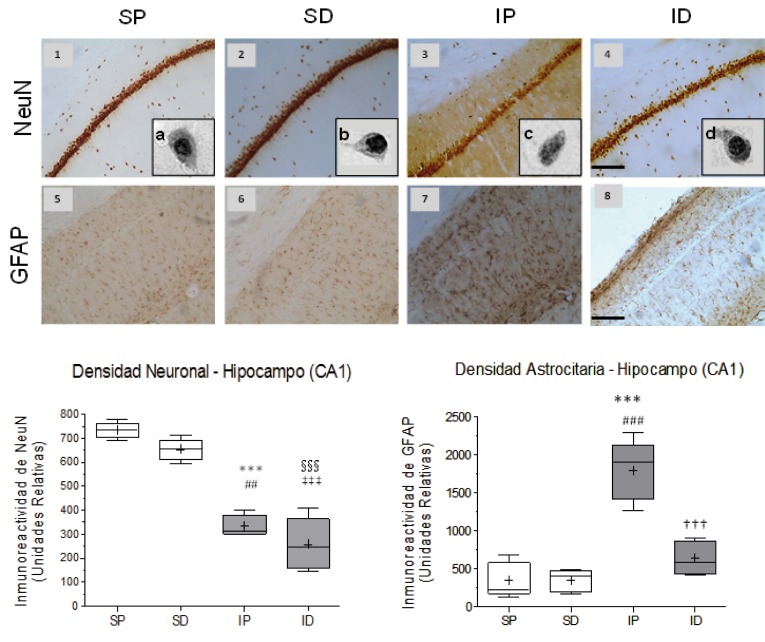
Effect of cerebral embolism and DMSO treatment on neuronal and astrocytic reactivity in the hippocampal CA1 area. A) Representative images of neuronal nuclear immune reactivity (NeuN) (1-4) and of the astrocytic immune reactivity (GFAP) (5-8) from the groups: SP (Sham + Placebo), SD, (Sham + DMSO), IP (Ischemia + Placebo), and ID (ischemia + DMSO). A, b, c and d correspond to magnified images of individual cells of the hippocampal CA1 region of the respective groups (100X in gray scale). (d). Disintegration of the nuclear chromatin and of the surrounding cytoplasm (c) was found as an effect of the ischemia differing from the control (a, b). DMSO preserves neuronal structure and the nuclear integrity after the ischemic event (d). B) Statistic for the immune reactivity of NeuN. Highly significant differences were observed between the IP/SP groups (*** p <0.001), ID/SP (§ § § p <0.001), ID/SD (‡ ‡ ‡ p <0.001) and significant differences between the groups IP/SD (# # p <0.01). C) Statistic of the immune reactivity of GFAP. Highly significant differences were found between IP/SP groups (*** p <0.001), IP/SD groups (# # # p <0.001) and ID/IP groups († † † p <0.001). Data are expressed as the mean ± SEM. Scale bar = 50 microns.

The cerebral emboli drastically reduced the immunereactivity of NeuN in the somatosensory cortex and ipsilateral hippocampus (CA1) Fig. 1. (1A3, 1B,) fig. 2. (2A3 and 2B) with significant differences between the ischemia placebo group (IP) and sham placebo groups (SP) (*p <0.05) and sham DMSO (SD) (# *p* <0.05) in the cortex, with highly significant differences between the IP group and its control SP (*** *p* <0.001) and between the IP group and SD group (## *p* <0.01) in CA1. The immune reactivity of neurons in the ischemic group treated with DMSO (ID), was similar to the ischemia group placebo (IP) in the cortex and hippocampus Fig. 1. (1A4 )and fig. 2. (2A4) In the hippocampus (CA1) nuclear chromatin condensation and neuronal shrinkage were observed Fig 2. (2A3 - block c) due to ischemia in comparison with the SP control Fig. 2.( 2A1 - block a). Treatment with DMSO was not able to totally reverse the loss in neuronal immune reactivity induced by the embolism Fig. 1. (1B) and fig. 2 (2B), although the damage was less in both the cortex (1A4) and the hippocampus Fig. 2 (2A4). However, nuclear integrity was maintained along with cellular morphology Fig. 2. (2A4 block d) similar to the control groups Fig. 2. (2A1a and 2A2B).

As to the response of astrocytes to the cerebral embolism, hyper-reactivity was evident in the somatosensory cortex Fig. 1. (1A7) and hippocampal CA1 Fig 2. (2A7) as compared with non-ischemic controls Fig. 1. (1A5-6 ) and Fig. 2. (2A5-6), with highly significant differences in the IP group in relation to the SP group (*** *p* <0.001) and the SD group (# # # *p* <0.001) as shown in Fig. 1. (1C) and Fig. 2. (2C).

**Figure 3:**
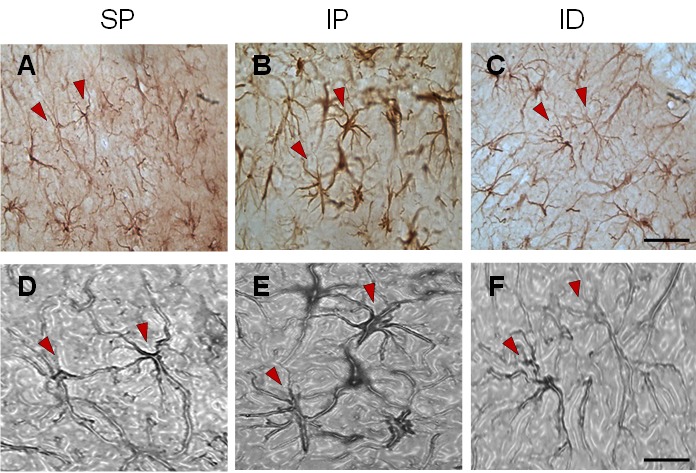
Representative microphotographs of astrocytic reactivity in the hippocampus of rats subjected to multifocal ischemia by arterial embolization and the effect of DMSO. A) Field with typical astrocytes from the sham + placebo group (SP) apparently normal, characterized by thin cell bodies and long, highly branched cytoplasmic processes. B) Astrocyte types from the ischemia + placebo (IP) group with shortening of cytoplasmic projections, thickening of the cell body and evident increase in internal density, and morphological changes characteristic of post-ischemic astrocytic hyper-reactivity process. C) Astrocyte types from the sub-granular zone of the hippocampus following treatment with DMSO in which long cytoplasmic projections, thin, highly-branched cell bodies were observed similar to the control group (SP).D, E and F correspond to plasticized images, magnified and in a gray scale that were obtained from the original photographs (A, B and C) respectively. The arrowheads show typical astrocytes in each of the states: normal (A and D), hyper-reactive characteristic of ischemia (B and E) and recovered from treatment with DMSO (C & F). Scale bar of C = 50 microns for the pictures in the upper row and 20 microns in F for images in the lower row.

Hyper-reactivity was characterized by a thickening of the cell bodies, shortening of cytoplasmic processes and increased density of the GFAP protein Fig. 3. (3B and 3E). 

In the ischemic group treated with DMSO, astrocytic hyper-reactivity was drastically reduced in the cortex and ipsilateral hippocampus (CA1) (see Fig. 1A8 and 2A8) in relation to the IP group, with highly significant differences (T T.T *p* <0.001) as observed in Fig. 1 (C) and Fig. 2(C). In the ID group, morphological restoration of the astrocytes is evident Fig. 3 (C, F) toward forms similar to the non-ischemic controls Fig. 3 (A, D), with equally long astrocytic processes and thin cellular bodies.

## Discussion

In this study a distribution pattern of lesions was observed similar to that described by other authors with the multifocal model for strokes15 and temporary focal occlusion of the MCA ^4^ ^,^ ^17^ ^,^ ^18^ Although other areas, such as the corpus callosum , hippocampal CA2-CA3, and the thalamic nuclei and striatum were studied, changes were evidenced only in the somatosensory cortex and hippocampus (CA1). In other experiments carried out by our group with the arterial embolization model, infarct distribution was corroborated by staining with triphenyl tetrazolium chloride (TTC), particularly engaging the cortex and ipsilateral hippocampus and in some cases the lateral striatum body (data not shown).

In the infarct zone, there is a drastic reduction in the immunoreactivity of NeuN, this being a sensitive marker of neurodegeneration wherein neuronal nuclear protein expression increases in the peri-infarction area and reduces in the ischemic nucleus ^19^. Our work is consistent with this observation where neuronal immunoreactivity is severely decreased in the cortical and hippocampal infarcted areas Fig. 1 (A3) and Fig. 2(A3). In another study of I/R in rat cerebrum, nuclear chromatin condensation and neuronal shrinkage in the hippocampal CA1 was observed after 7 days ^20^, which is similar to our results where significant morphological changes and density of the nuclear substance was evidenced Fig. 2(A) and Fig. 3(c). Similarly, necrotic cell death has been reported accompanied by irreversible changes in the nucleus, such as karyolysis, pyknosis and karyorrhexis ^21^ as well as the loss of structure and fragmentation of the cytoplasm, similar to what is observed at high magnification Fig. 2 (A) and Fig. 3(c) when compared with the non-ischemic control Fig. 2(A) and Fig. 1(a).

Possibly the astrocytic hyper-responsiveness observed in this study corresponds to a reactive gliosis given the course of ischemia (13 days) being in agreement with other studies in which, after seven days of I/R, reactive gliosis was evident in hippocampal CA120 and most strikingly in the peri-infarction areas ^22^.

It has been described that in rats only eight minutes of vascular occlusion induces a selectively delayed neuronal death in the hippocampal CA1. Transient ischemia induces activation of astrocytes in early stages; however, proliferation is much less and more delayed. Activated astrocytes adopt a hypertrophic morphology but do not increase significantly in number and proliferate later (2-5 weeks post I / R) in the chronic stage, which is crucial for the tissue restoration stage by the release of neurotrophic substances and antioxidants that aid survival ^22^. It is likely that at two weeks post-ischemia astrocyte proliferation is not yet present, which is characteristic of a more chronic course.

In our study, the ischemia induced by cerebral embolism showed a significant decrease of neuronal immunoreactivity in the cortex and hippocampus (CA1) while the astrocytic hyper-reactivity was maximal. This suggests an inverse relationship between the number of NeuN immunoreactive neurons and the number of astrocytes at the same time in the ischemic course, similar to that described in the hippocampal CA4 area with moderate neuronal loss and hyper-reactive astrocytes ^6^.

The present study corresponds to the early stage of neuronal and astrocytic response demonstrating a drastic reduction in astrocytic hyper-reactivity as an effect of DMSO, although this failed to reduce the loss of neuronal immunoreactivity. At this stage, the astrocytes become reactive with extensive nuclear hypertrophy of the cell body and cytoplasmic processes and increase of immunodetectable glial fibrillary acidic protein (GFAP) ^23^. Possibly in the early phase of brain injury, the immunoreactivity of GFAP is a key marker of astrocyte hyperactivity and tissue damage.

In our model, the administration of DMSO at a dosage of 450 mg/kg (100 μl at 90%) significantly reduced astrocytic hyper-reactivity post-ischemia and although the neuro-protective effect of DMSO has been described as a capturer of free radicals, its mechanism of exact action is still unknown along with its cellular and molecular targets in the CNS.

Moreover, the integrity of the BBB and the functionality of its transport system are crucial to the proper functioning of the CNS ^18^. DMSO has been studied in combination with diphenyl eneiodonium (DPI), a free radical inhibitor, where focal cerebral post-ischemia neuro-protective properties were observed in rats. The reduction in lesion size as a result of potentiated neuro-protective effect is attributed to the nonspecific capture of free radicals and the inhibition of metalloproteinases, which reduces damage to the BBB and improves the neurologic response ^24^.

Based on the results obtained, it is possible that DMSO administered in combination with other neuro-protectors improves neurological response, reduces infarct volume and neuronal loss, in addition to controlling astrogliosis that leads to delayed neuronal and glial death.

Knowing the involvement of astrocytes in ischemia from cerebral embolism may help elucidate the intrinsic capacity of CNS to confront the ischemic insult and to identify new molecular targets for therapeutic purposes.

## Conclusions

DMSO significantly reduces astrocytic hyper-reactivity (gliosis) in the areas of the cerebrum affected by arterial embolization and, although it does not diminish the loss of neuronal immune reactivity, it prevents the changes in cytoarchitecture that might be related to neurotoxicity.The regulatory effect of DMSO on astrocytes and neurons after arterial embolization potentially confers neuroprotective properties for the treatment of thromboembolic cerebral ischemia in the acute phase.
